# IQSEC2‐related encephalopathy in males due to missense variants in the pleckstrin homology domain

**DOI:** 10.1111/cge.14136

**Published:** 2022-04-06

**Authors:** Cheryl Shoubridge, Tracy Dudding‐Byth, Laurent Pasquier, Himanshu Goel, Patrick Yap, Vivienne McConnell

**Affiliations:** ^1^ Robinson Research Institute, and Adelaide Medical School University of Adelaide Adelaide Australia; ^2^ Hunter Genetics Waratah Australia; ^3^ CHU Rennes, Service de Génétique Clinique Centre de Référence Déficiences Intellectuelles Rennes France; ^4^ Genetic Health Service New Zealand (Northern Hub) Auckland New Zealand; ^5^ Northern Ireland Regional Genetics Service Belfast City Hospital, Belfast Health & Social Care Trust Belfast UK

**Keywords:** epilepsy, *IQSEC2*, pathogenic sequence variant, PH domain

## Abstract

Pathogenic variants in IQ motif and SEC7 domain containing protein 2 (*IQSEC2*) gene cause a variety of neurodevelopmental disorders, with intellectual disability as a uniform feature. We report five cases, each with a novel missense variant in the pleckstrin homology (PH) domain of the IQSEC2 protein. Male patients all present with moderate to profound intellectual disability, significant delays or absent language and speech and variable seizures. We describe the phenotypic spectrum associated with missense variants in PH domain of *IQSEC2*, further delineating the genotype–phenotype correlation for this X‐linked gene.

## Graphical abstract

We report five novel missense variants in the pleckstrin homology (PH) domain of the IQSEC2 protein. Male patients all present with moderate to profound intellectual disability, significant delays or absent language and speech and variable seizures, consistent with the features in other reported cases and highlights the importance of the PH domain for IQSEC2 function.

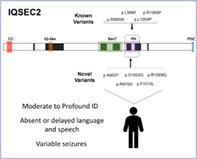

## 1 INTRODUCTION

The IQ motif and SEC7 domain containing protein 2 (IQSEC2) is encoded by the *IQSEC2* gene [NM_001111125] (MIM# 300522), which spans a 93.7 kb genomic region on chromosome X at Xp11.22. The IQSEC2 protein catalyses exchange of GDP for GTP in several of the ARF superfamily of proteins and has an essential role in modulating the cytoskeleton and vesicle transport at the post‐synaptic density and hence is a crucial modifier of synaptic plasticity.^1^

We and others have identified disease‐causing variants in *IQSEC2* that invariably cause intellectual disability (ID), and frequent severe early‐onset seizures.^2^, ^3^, ^4^, ^5^ Here, we report five cases of affected males with moderate to profound ID, seizures and speech and language disorders likely due to novel missense variants in the pleckstrin homology (PH) domain of *IQSEC2*. This domain recruits proteins to membranes via interactions with phosphoinositide, targeting the ArfGEF to appropriate cellular compartments and signal transduction pathways.^6^ We review the growing number of published cases of missense variants in PH domain and highlight the largely consistent phenotypes of ID, epileptic encephalopathy with speech and language deficits.

## 2 MATERIALS AND METHODS

See Data S1.

## 3 RESULTS

### 3.1 Case ascertainment, patient phenotype, variant detection, and clinical interpretation

We present five nonsynonymous missense variants that impact the PH domain of the I*QSEC2* gene that have not been previously reported (Table 1). Detailed clinical description and screening outcomes for Family 1 to 5 is described in Data S1 and presented in Table 1.

**TABLE 1:** Clinical features of patients

*IQSEC2* variant	Patient (FAM) 1	Patient (FAM) 2	Patient (FAM) 3	Patient (FAM) 4	Patient (FAM) 5
NM_001111125.2 NP_001104595	c.2857G > A p.(Ala953Thr)	c.2909G > A p.(Arg970His)	c.3005A > G p.(Asp1002Gly)	c.3030C > G_p.(Phe1010Leu)	c.3206G > A p.(Arg1069Gln)
Gr37 (hg19) Exon	chrX:53272546 Exon 9	chrX:53271072 Exon 10	chrX:53270976 Exon 10	chrX:53268462 Exon 11	chrX:53267398 Exon 12
Inheritance	unknown	Familial	Maternal	unknown	unknown
Novel (CS)	Novel	Novel	Novel	Novel	Novel
ACMG classification	Likely pathogenic	Likely Pathogenic	Likely Pathogenic	VUS	VUS
CADD	23.5	35	29.8	22.8	35
Gender	Male	Male	Male	Male	Male
First diagnosis (years)	—	—	—	12 months	4 months
Last follow‐up (years)	31	12	8	3.5 years	16 years
Race	Chinese	French Caucasian	Caucasian	Mixed Maori Caucasian	Caucasian
Main clinical features/diagnosis	Non‐verbal severe ID	Severe ID + Drug resistant seizures LGS and ID	Dystonic quadriplegic CP, epilepsy, severe ID, poor wt gain	Global DD, multiple congenital anomalies, distinctive facial gestalt Undiagnosed NDD syndrome	Severe intractable epilepsy (EIEE) with onset at 4 months and significant global DD
Seizures	Yes	Yes	Yes	None documented	Yes
Age at onset	15 years	4 years	6 months		4 months
Seizure types	Generalised seizures	abnormal EEG no clinical symptoms	Myoclonic jerks, probably infantile spasms		EIEE and subsequently tonic–clonic, intractable
Epileptic syndrome		LGS	EE		EIEE
Speech & language	non‐verbal	Absent speech	profound language delay	significant delay	Almost non‐verbal
Onset/regression	2 years			3 years 6 months	No regression
Achievements	Communicates by pointing to pictures, understands simple conversations and instructions	Sounds and chattering tongue	Incomprehensible sounds	Using single words. Gestures to indicate wants/dislike. Knows colours, names and numbers	2 single words currently at 16 years – “mum” and “boom”
Development		No toilet‐training Restricted autonomy	Severe to profound delay in all domains	Globally delayed, 3.5 years	Profound severe global DD
Initial motor development	Normal	Able to walk at 2 years	Hypertonia, severe delay	Delayed from early infancy, torticollis in infancy	Holding head up at 2 years crawling at 2.5 years walking at 4–5 years
Regression‐age	2 years	No regression	9 months	No regression	N/A
Intellectual disability	Moderate to severe	DD, Severe ID	Global DD, severe to profound ID	Global DD ID not formally assessed	Significant global DD, Severe profound ID Special school
Behavioural anomalies Autistic features Stereotypies	Aggressive, self‐mutilation ASD	ASD stereotypies	Constantly sucking on his hands.		Requires 24/7 supervision Autistic behaviour Stereotypical movement
MRI		Normal at 2 and 11 years	Non‐specific decrease in white matter volume	Not done	Congenital microcephaly (>0.4th) (2 years) generalised cerebral atrophy/hypoplasia. (7 years) bilateral mesial temporal sclerosis
Other features not captured above		Able to walk without ataxia Not dysmorphic			Short stature 0.4th–2nd centile weight 2nd centile, drooling, pyloric stenosis, dysmorphic, doubly incontinent – wears nappies

*Family 1*: The patient is a 31‐year‐old male with moderate to severe ID, autism spectrum disorder (ASD), with generalised seizures. Although non‐verbal, he communicates by pointing at pictures and can understand simple instructions. A molecular karyotype shows a small 6q11.1q12 duplication (0.86 Mb) of uncertain significance intercepting the *KHDRBS2* gene, which is not known to be associated with ID. Whole‐exome sequencing detected a hemizygous variant in *IQSEC2*, c.2857G > A, p.(Ala953Thr). The ClinVar variant identity is 1 321 155. Parental studies are unavailable. His sister and maternal uncle do not carry this *IQSEC2* variant.

*Family 2*: At 2 years of age the patient started to walk but had global developmental delay and absent speech. He was subsequently diagnosed with a severe neurodevelopmental disorder including an ASD with stereotypies, absent speech and ID. Worsening seizures lead to a diagnosis of Lennox–Gastaut syndrome. Exome sequencing detected a hemizygous variant in *IQSEC2*, c.2909G > A, p.(Arg970His). The LOVD variant identity is 832 180. This variant was maternally inherited but de novo in the mother.

*Family 3*: The patient had global developmental delay from 5 months of age. There were head nodding and hyperextension episodes at 10 months of age, and he later developed myoclonic jerks, hypertonia and brisk reflexes. His EEG showed hypsarrythmia and a diagnosis of epileptic encephalopathy was made. Whole exome sequencing showed a hemizygous variant: *IQSEC2*, c.3005A > G, p.(Asp1002Gly). The ClinVar variant identity is 1 321 179. This variant was maternally inherited.

*Family 4*: The proband was born at term by a normal delivery with multiple congenital abnormalities including rib and vertebral segmentation anomalies, L1 dysplasia, bilateral undescended testes, and right inguinal hernia. Global developmental delay is evident from late infancy. Whole exome sequencing detected a hemizygous variant: *IQSEC2*, c.3030C > G, p.(Phe1010Leu). The Decipher variant identity is 453 200. Maternal DNA is not available for testing at time of publication.

*Family 5*: This patient has profound global developmental delay, is almost nonverbal with 1–2 abbreviated words and received a diagnosis of severe epileptic encephalopathy at 4 months of age. At 16 years of age, he has severe to profound ID, autistic spectrum disorder, behavioural and sleep difficulties. A molecular karyotype, involving an Agilent (ISCA v2) 8x60K oligo array platform identified an interstitial duplication involving chromosome 6q14.2 with minimum and maximum size of 400 and 600 kb, respectively which includes few genes including one MIM Morbid gene, RIPPLY2 (MIM 609891) and has not been maternally inherited (no paternal DNA sample available) and considered unlikely to be contributing to patient's phenotype. Whole exome sequencing detected a hemizygous missense variant in *IQSEC2*, c.3206G > A (p.Arg1069Gln). Parental studies are unavailable. The LOVD variant identity is 832 181.

To achieve a consistent clinical interpretation of genetic variation by ACMG/AMP 2015 guidelines,^7^, ^8^ we utilised wIntervar,^9^ see Data S1 in conjunction with the various clinical teams supporting each family. Variant assessment using the prediction tool CADD^10^, ^11^ (CADD score of or 20 or above indicates a variant is amongst the top 1% of deleterious variants in the human genome) are shown for each variant in Table 1. The reduced tolerance to variation of the PH domain is shown on Table S1.

ID, seizure, autism and severe speech and language deficits phenotypes due to missense mutations in the *IQSEC2* PH like domain.

Including the five novel variants that cause amino acid changes reported here there is a total of 9 different nonsynonymous missense pathogenic variants in the *IQSEC2* PH domain, with two reported in affected females (Table 2). The variant in Family 5 in this study (p.Arg1069Gln) impacts the same amino acid residue in a previously reported patient (Patient 33, p.Arg1069Pro),^3^ both with strikingly similar phenotypes (Table 2). Despite the consistent phenotypes we observe that the responses to antiepileptic treatments were variable, regardless of the treatment (Table 2).

**TABLE 2:** Response to treatment for seizures in patients with missense variants in the PH domain of *IQSEC2*

Variant protein NP_001104595	Case	Sex	Phenotype						Reference
			DD/ID	Seizures	Drugs tried + response to therapy		Speech deficits	Behavioural/psychiatric/physical	
					No improvement	Improvement/management			
p.Ala953Thr	FAM 1	M	Severe ID	Generalised seizures		Combination therapy clonazepam, sodium valproate and phenytoin	Regression in speech Non‐verbal	Aggressive, ASD, self‐mutilation	This study
p.Arg970His	FAM 2	M	DD/Severe ID	LGS	Clobazam (4 years), then zonisamide, topiramate, ethosuzimide	Rufinamide and VNS therapy	Absent speech	ASD, stereotypies, restricted autonomy. Normal brain MRI	This study
p.Arg995Trp	P7	F	Global DD, ID	No			Regression in language	Hypotonia (Rett‐like)	12
p.Leu999Phe	P5	M	DD/Severe ID	Early‐onset epilepsy	Valporic acid	Clobazam and topiramate tolerated but breakthrough seziures, Ketogenic diet	Virtually non‐verbal	Non‐ambulatory, self‐harming. MRI: generalised volume loss	13
p.Asp1002Gly	FAM 3	M	Global DD Severe‐profound ID	EE	ACTH, Vigabatrin Partial response – Nitrazepam, biotin and folic acid	Topiramate and Keppra	Profound language delay	Hypotonia, MRI shows non‐specific decrease in white matter volume	This study
p.Leu1004Pro	P11	F	Severe ID	Localised tremors, tonic–clonic crises	N/A	N/A	Absent speech	Autism, self‐harming, stereotypies	14
p.Phe1010Leu	FAM 4	M	Global DD	None			Significant speech and language delay	Multiple congenital anomalies and distinctive facial gestalt	This study
p. Arg1069Pro	P33	M	Severe ID	EE	Sodium valporate	Clonazepam, Lamotrigine	Non‐verbal	Autistic behaviour	4
p.Arg1069Gln	FAM 5	M	Global severe profound DD and ID	Intractable epilepsy	Phenobarbitone, carbamazepine, clobazam, clonazepam, sodium valproate, gabapentin, lamotrigine, phenytoin, topiramate, vigabatrin, zonisamide, levetiracetam, pyridoxine	Combined THC/CBD and Lacosamide for previous 2 years and only seizure during this period requiring hospitalisation	Almost completely non‐ verbal‐	ASD, stereotypic movements, dysmorphism, microcephaly MRI abnormalities	This study

Abbreviations: ASD, autistic spectrum disorder; DD, developmental delay; EE, epileptic encephalopathy; ID, intellectual disability; LGS, Lennox–Gastaut syndrome; N/A, not available/assessable.

*Note*: Amino acid numbering reflects reference sequence for the IQSEC2 protein [GenBank: NP_001104595]. Blue, male; Pick, Female

### 3.2 In‐silico analysis of PH protein variation

**FIGURE 1:**
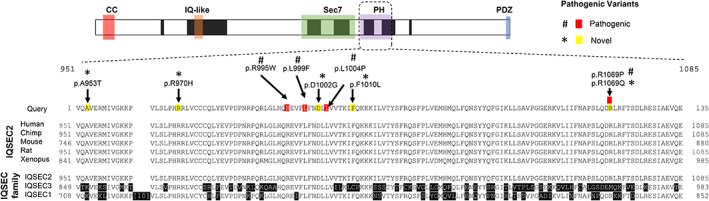
Known and novel missense variants in the PH domain of *IQSEC2* leading to ID, variable seizures and severe speech and language deficits. (A) The predicted protein structure (NP_001104595) with known functional domains highlighted, coiled‐coiled (CC, red), IQ‐like (orange), Sec7 enzyme domain (Sec7, green), PH domain (purple) and the PDZ binding motif (blue). Amino acid residues p.951 to p.1085 of the PH domain are shown. Conservation of the PH domain in the IQSEC2 protein across different species (Human NP_001104595, Chimp PNI19549, Mouse NP_001108136, Rat NP_001264354, Xenopus XP_002941016) and across the IQSEC protein family (IQSEC1 NP_001127854, IQSEC3 NP_001164209). Black highlight indicates variable residues compared to the IQSEC2 reference. Clinically relevant pathogenic missense variants are indicated in residues (red, hash). Novel missense pathogenic variants reported in this study are indicated in a yellow (star) [Colour figure can be viewed at wileyonlinelibrary.com]

The longest isoform of IQSEC2 (NP_001104595.1) is the dominant transcript expressed in the brain. The sequence of the PH domain of IQSEC2 is conserved across species (down to amphibians) (Figure 1). When we searched the human genome for other proteins with similar PH domains, we identified only the IQSEC family members with substantial homology (Table S2). The location of the missense variants in the PH domain of IQSEC2 spread across the ~135 amino acid domain are shown in reference to conserved and related IQSEC1 and IQSEC3 proteins (Figure 1).

## 4 DISCUSSION

There is an emerging clinical picture of ID, epileptic encephalopathy with speech and language deficits and autism due to a broad range of variants in the *IQSEC2* gene, including the missense variants in the PH domain that we report here. When we include the five novel cases presented in this study, there are 29 distinct nonsynonymous missense variants in the *IQSEC2* gene reported in 34 unrelated cases and families.^2^, ^3^, ^12^, ^14^, ^15^, ^16^, ^17^, ^18^, ^19^, ^20^ Most of these variants occur in known functional domains. Within the catalytic Sec7 domain, nine missense variants with three recurrent variants across eight separate cases or families, impact a total of 44 affected individuals (39 males and five females). There are (4/29) missense variants within the IQ‐like domain (all male) and (7/29) outside a known functional domain (four males and three females). Although the reporting of clinical features for patients is not always consistent or complete (particularly for affected females), mild–moderate through to severe‐profound ID is a consistent finding, with 30% of patients reported with seizures, 15% with speech and language deficits and 20% with ASD features. Carrier females were also reported in several large families, often with borderline ID, learning difficulties or mild ID.

There are nine disease‐causing missense variants in the PH domain, including the five cases reported in this study. The phenotypes range from affected females with either Rett‐like features with language regression,^12^ absent speech and language, ASD and severe ID^14^ through to affected males with severe non‐verbal ID and early‐onset epilepsy.^4^, ^13^ The cases reported in this study had moderate to profound ID (5/5), seizures (4/5) and significant delays or absent language and speech (4/5), consistent with the phenotype.

The phenotype severity highlights the importance of the PH domain for IQSEC2 function, while the largely consistent spectrum of features indicates that these variants are having a similar impact on the function of this domain. It is well established that PH domains are a major type of membrane binding domain whose structure and sequence provides insights into their biological functions. Despite high variability in the homology of PH domains across different classes of proteins, the amino acid sequence of the PH domain is highly conserved within the IQSEC family. ArfGEFs with PH domains including cytohesins, EFA6 and IQSEC subfamilies at plasma membranes are highly potent in activating Arfs, but each via a different allosteric mechanism.^21^

The crystal structure of IQSEC1 in complex with Arf shows the PH domain forms extensive intramolecular interactions with the Sec7 domain, with high affinity binding of phosphatidylinositol 4,5‐biphosphate (PIP_2_) enhancing intrinsic GEF activity toward ARF6.^22^, ^23^ Despite the phosphoinositide binding specificity for IQSEC2 remaining unknown, the crystal structure studies have shown that the binding occurs so that the GTPase and the PIP_2_ binding site interact with membranes. Hence, we contend that the variants occurring at the Sec7‐PH domain interface may impair the coupling of the IQSEC2 ArfGEF function with the membrane‐binding function. This hindering of allosteric regulation on membranes constitutes a rational mechanism of pathogenesis. Future work is warranted to dissect how variants in the PH domain of IQSEC2 interferes with the protein activity and determine how dysfunction in this domain leads to early‐onset epileptic encephalopathy and ID in affected individuals.

## AUTHOR CONTRIBUTIONS

*Contributed the patient and clinical data for Family 1, Family 2, Family 3, Family 4, Family 5, respectively*: Tracy Dudding‐Byth, Laurent Pasquier, Himanshu Goel, Patrick Yap, Vivienne McConnell. *Performed in silico analysis*: Cheryl Shoubridge. *Wrote the first draft of the manuscript, with all authors contributing to discussion of the results and final manuscript preparation*: Cheryl Shoubridge.

## CONFLICT OF INTEREST

The authors declare they have no competing interests.

### PEER REVIEW

The peer review history for this article is available at https://publons.com/publon/10.1111/cge.14136.

## Supporting information


**Appendix** **S1:** Supporting Information

**Table** **S1:** Pathogenic and tolerated variation in the PH domain of IQSEC2.

**Table** **S2:** Conservation of functional domain structure in ArfGEF family.

## Data Availability

All data generated or analysed during this study are included in this published article [and its supplementary information files]. Data sharing is not applicable to this article as no new data were created or analyzed in this study.
