# Supplementary material for: IQSEC2‐related encephalopathy in males due to missense variants in the pleckstrin homology domain
Source: Clin Genet. 2022 Apr 6;102(1):72–7. doi: 10.1111/cge.14136 (PMC9325495; doi:10.1111/cge.14136)
Supplement: Supplementary file 1 — Appendix S1: Supporting Information [file CGE-102-72-s001.docx]

**Supplementary Data file: Data S1**

**Materials and methods**

**Phenotype of patients**

The relevant research ethics committees and institutional review boards of collaborating institutions approved this study. Clinical information and DNA samples were collected with informed consent.

***Family 1*:** The patient is a 31-year-old male with moderate to severe ID, autism spectrum disorder (ASD), with generalised seizures. He was the firstborn to unrelated Chinese parents and was delivered at full term following an uncomplicated pregnancy with no teratogenic exposure. He reached his early developmental milestones, but his speech regressed around two years of age, and he became increasingly aggressive. His behaviour includes hand flapping and rocking, and he was subsequently diagnosed with ASD. He developed generalised seizures controlled with a combination of clonazepam, sodium valproate, and phenytoin. Although non-verbal, he communicates by pointing at pictures and can understand simple instructions. He can feed, dress and toilet himself but needs supervision in the group home setting. He can become agitated with a change in routine, but his aggressive behaviour, autistic traits and epilepsy are well controlled with medication. Self-mutilation remains a constant concern. A maternal uncle with mild learning problems and bipolar disorder lives independently, but there is no family history of ID. His height is 168 cm (10^th^ – 25^th^ centile) with a weight of 75 kg (75^th^ centile). He would not allow his head circumference to be measured. Clinical features included a prominent jaw and scarring from self-mutilation. Normal investigations include fragile X, urine metabolic screen and 15q11.2-q13 methylation studies.

***Family 2*:** The patient was the second child of healthy non-consanguineous parents and a healthy sister. He was born at 42 weeks gestation without complication (no diabetes). At birth, weight was 4700 gm, length 52 cm, and OFC 36 cm with Apgar scores of 10 and 10 at 1 and 5 mins. At two years of age, he started to walk but had global developmental delay and absent speech. He was subsequently diagnosed with a severe neurodevelopmental disorder including an ASD with stereotypies, absent speech (only sounds and chattering tongue) and ID. Currently, he shows limited autonomy and is not yet toilet-training. At four years of age, an abnormal EEG was identified without clinical symptoms, and clobazam therapy was implemented. Worsening seizures lead to a diagnosis of Lennox-Gastaut syndrome. His seizures were resistant to zonisamide, topiramate and ethosuximide but improved with rufinamide and vagus nerve stimulation (VNS). Brain MRI scan was performed at 2 and 11 years of age and showed no abnormalities. At 12 years of age, a growth retardation was detected: height 128 cm (<1^st^ centile), weight 22.5 kg (-2 DS) IMC 13.4 kg/m². His array CGH and serum transferring isoforms were normal.

***Family 3*:** The patient was seen by a paediatrician at 12 months of age with concerns about his global developmental delay. He is the second of three children, and both parents have some learning problems. The mother’s maternal half siblings also have learning problems. Family studies are unavailable. The patient was born at full term via a normal delivery with birth parameters < 10^th^ percentile. He was developing normally until five months of age, at which stage his developmental progress stagnated. There were head nodding and hyperextension episodes at 10 months of age, and he later developed myoclonic jerks, hypertonia and brisk reflexes. His EEG showed hypsarrythmia and a diagnosis of epileptic encephalopathy was made. His brain MRI at 12 months of age showed a nonspecific decrease in white matter volume. Mutation screening for *ARX* was negative and *MECP2* MLPA/sequencing was normal. White cell enzymes and plasma glucosylsphingosine were also normal. At eight years of age his head circumference was 2cm under 2^nd^ percentile, height was 5cm under 1^st^ percentile and his weight was on the 3^rd^ percentile.

***Family 4*:** The proband is the firstborn male infant to non-consanguineous couple of mixed Maori-Caucasian descent. He was born at term by a normal delivery with a birth weight of 3.96 kg and multiple congenital abnormalities. He was managed in the NICU for respiratory distress syndrome. Congenital anomalies include rib abnormalities (13 ribs on the right, 11 ribs on the left), vertebral segmentation anomalies involving C1-C3 and T4-T5, L1 dysplasia, bilateral undescended testes, and right inguinal hernia. There was torticollis, which resolved with physiotherapy, and no evidence of hip dysplasia. Global developmental delay is evident from late infancy. A developmental assessment at 3 years 6 months estimated a developmental age of 24-30 months for gross motor and 18-24 months for fine motor, and speech and language skills. There are no features of ASD, seizures or regression.

The proband has distinctive craniofacial features, characterised by brachycephaly, flat facial profile, arched eye-brows, down-slanting palpebral fissures, long eye-lashes, prominent nose, long philtrum and thin upper lip. He has generous earlobes with no pits or creases. He has hypertrichosis on the upper back and limbs. He has hypoplastic nails of the index fingers and toes. Growth parameters were normal.

The variant identified in this patient has not been specifically looked for in other Maori patients. Given that multiple congenital anomalies are not normally associated with IQSEC2-related disorders and the lack of overt seizures there is no alternate diagnosis. A re-analysis of the exome did not indicate an alternative diagnosis.

***Family 5*:** This patient was born by an emergency caesarean section at 34 weeks gestation with a birth weight of 1.87kg (10-50^th^ percentile) due to placenta praevia and is the only child of non-consanguineous Caucasian parents. He has profound global developmental delay, holding his head up at two years of age, crawling from 32 months of age, and walking at 4-5 years of age. He did not have a history of developmental regression. Currently, he is almost nonverbal with 1-2 abbreviated words. A diagnosis of severe epileptic encephalopathy was made at four months of age. At 16 years of age, he has severe to profound intellectual disability, autistic spectrum disorder, behavioural and sleep difficulties.

Generalised cerebral atrophy was first detected on brain MRI at 2 years. MRI brain scan at seven years of age confirmed bilateral diffuse cortical atrophy predominantly in frontal and temporal regions. Intractable tonic-clonic seizures are reasonably controlled with lacosamide, cannabidol CBD /THC combination. His current height is at 2^nd^ percentile, weight is at 10^th^ percentile and his head circumference is less than 0.4^th^ percentile.

He had corrective surgery for congenital hypertrophic pyloric stenosis as a neonate. There is family history of epilepsy in a maternal great aunt and of developmental delay and epilepsy in a maternal nephew whose clinical phenotype is not felt to be similar. His array CGH was noninformative. Methylation studies of Prader Willi Syndrome and Sanger sequencing of *ARX*, *SCN1A*, *CDKL5*, *STK9*, *MECP2* were normal. His 48 gene epilepsy and hepatocerebral mitochondrial DNA panels were also normal.

**Molecular analyses**

The appropriate institution review boards approved the screening protocols, and informed consent was obtained from the parents of patients. The study conforms with the principles set out in the WMA Declaration of Helsinki and Australian National Statement on Ethical Conduct in Human Research (2018).

The DNA from the affected patient of Family 1 was screened by Fulgent Genetics (Temple City, CA) using the ID NGS panel (495 genes sequencing and deletions/duplication). The DNA from the affected patient and his parents (Trio-based family) of Family 2 was whole-exome sequenced (WES) on an Illumina MiSeq platform located in the Rennes University Hospital Genetic Lab. The DNA from the affected patient of Family 3 was screened by whole exome sequencing of an epileptic encephalopathy gene panel. For the proband in family 4 a chromosomal microarray (SNP-array) did not detect any clinically significant imbalances and Fragile X testing was negative. The proband proceeded to singleton whole exome sequencing through a commercial clinical laboratory. Family 5 was enrolled in Deciphering Developmental Disorders (DDD), UK research. The *IQSEC2* gene missense variant was identified using whole exome sequencing. The DDD Study ([www.ddduk.org/access.html](http://www.ddduk.org/access.html)) presents independent research commissioned by the Health Innovation Challenge Fund [grant number HICF-1009-003]. This study makes use of DECIPHER ([www.deciphergenomics.org](http://www.deciphergenomics.org)), which is funded by Wellcome (DDD Study PMID: 25533962). Variants in I*QSEC2* (NM_001111125.2, NP_001104595) are reported against GRCh37/hg19 assembly.

**In-silico analysis of the PH domain protein variation**

The amino acid sequence of human IQSEC2 PH like domain (p.951 to p.1085) (NP_001104595.1) was used to identify proteins with homologous regions using the blastp algorithm against a non-redundant protein sequence on the Protein Basic Local Alignment Search Tool (BLAST) from NCBI. The resulting list was manually curated to avoid redundancy and ranked based on sequence homology with the IQSEC2 PH like domain.

To interrogate publicly available in silico prediction tools, each nonsynonymous missense variant, the chromosome, reference and alternate sequence and genomic coordinates were compiled into a vcf input file and analysed via the “all annotation” tool using wANNOVAR (Chang and Wang 2012, Wang *et al*., 2010).

**Clinical interpretation of variants**

The variants described in Family 1 to Family 4 were not present in the known single nucleotide polymorphism database GnomAD. The variant in Family 5 was present in GnomAD in a single heterozygous female (version V2.1.1) and in a single heterozygous female and a hemizygous male in V3.1 (Supplementary Table 1). We submitted the variants to clinical interpretation by the ACMG/AMP 2015 guidelines considering these variants are absent or at extremely low frequency in GNOMAD (PM2), located on the IQSEC2_PH domain (PM1) with manual adjustment to consider that the variants are predicted to have a deleterious effect based on computational evidence (PP3), and that *IQSEC2* has a low rate of benign missense variation and that missense variants are a common mechanism of disease in this gene (PP2). This InterVar adjusted classification identified that each of the variants would be considered as Likely Pathogenic. Despite this, clinical teams may choose to interpret and apply the guidelines with differing levels of caution. The clinical team for patient in Family 4 continue to consider the variant as a VUS given the atypical manifestation for IQSEC2-related disorder, and the clinical team for Family 5 consider this patients variant as a VUS.

Harnessing variant aggregation data from gnomAD database for *IQSEC2* indicates this gene has a reduced tolerance to variation with a constraint metric of z=5.19 for missense variants (expected 598, observed 241) and a pLi =1 for pLoF variants (expected 35.6, observed 1) (based on ensemble canonical transcript ENST00000396435.3). This reduced tolerance to variance is highlighted when we specifically focus on the nonsynonymous missense variants across the PH domain of IQSEC2 (Supplementary Table 1). Examining variants from both the V2.1.1 and non-V2 variants in the new V3 release, we see low numbers of variants, many as single cases, reflected by the low allele frequencies.

References

Deciphering Developmental Disorders Study. Large-scale discovery of novel genetic causes of developmental disorders. Nature. 2015 Mar 12,519(7542):223-8.

Chang, X., & Wang, K. (2012). wANNOVAR: annotating genetic variants for personal genomes via the web. J Med Genet, 49(7), 433-436.
