# Supplementary material for: IQSEC2‐related encephalopathy in males due to missense variants in the pleckstrin homology domain
Source: Clin Genet. 2022 Apr 6;102(1):72–7. doi: 10.1111/cge.14136 (PMC9325495; doi:10.1111/cge.14136)
Supplement: Supplementary file 2 — Table S1: Pathogenic and tolerated variation in the PH domain of IQSEC2. [file CGE-102-72-s003.docx]

|  | Position  GRCh37 | Transcript | Protein | Allele Count | Allele Frequency | Hemi | Het | ClinVar Sign | ClinVar  Variant ID | CADD >20 |
| --- | --- | --- | --- | --- | --- | --- | --- | --- | --- | --- |
| **Patients in this study** | | | | | | | | | | |
| Fam 1 | 53272546 | c.2857G>A | p.Ala953Thr |  |  | 1 |  | US | 1321155 | 23.6 |
| Fam 2 | 53271072 | c.2909G>A | p.Arg970His |  |  | 1 |  |  |  | 35 |
| Fam 3 | 53270976 | c.3005A>G | p.Asp1002Gly |  |  | 1 |  | LP | 1321179 | 29.8 |
| Fam 4 | 53268462 | c.3030C>G | p.Phe1010Leu |  |  | 1 |  |  | 453200  Decipher | 22.8 |
| Fam 5 | 53267398 | c.3206G>A | p.Arg1069Gln |  |  | 1 |  |  |  | 35 |
| **Published cases** | | | | | | | | | | |
| Sirvastava | 53270998 | c.2983C>T | p.Arg995Trp |  |  |  | 1 |  |  | 34 |
| Helm | 53270986 | c.2995C>T | p.Leu999Phe |  |  | 1 |  |  |  | 28.9 |
| Lopergolo | 53270970 | c.3011T>C | p.Leu1004Pro |  |  |  | 1 |  |  |  |
| Mignot | 53267398 | c.3206G>C | p.Arg1069Pro |  |  | 1 |  |  |  | 34 |
| **ClinVar** | | | | | | | | | | |
|  | 53270998 | c.C2983T | p.R995W |  |  |  |  | P/LP | 383534 | 34 |
|  | 53270997 | c.G2984A | p.R995Q |  |  |  |  | P/LP | 280590 | 35 |
|  | 53270986 | c.C2995T | p.L999F |  |  |  |  | LP | 810615 | 28.9 |
|  | 53267398 | c.G3206A | p.R1069Q |  |  |  |  | LP | 975246 | 35 |
| **Potentially benign variation** | | | | | | | | | | |
|  | GRCh37 |  |  |  |  |  |  |  |  |  |
| GnomAD V2.1.1 | 53272545 | c.2858C>T | p.Ala953Val | 1 | 8.6892E-06 | 0 | 1 |  |  | 23.2 |
|  | 53272536 | c.2867G>A | p.Arg956His | 2 | 1.7405E-05 | 1 | 1 | US | 104328 | 34 |
|  | 53271063 | c.2918T>C | p.Val973Ala | 1 | 5.6045E-06 | 0 | 1 |  |  | 28.3 |
|  | 53271024 | c.2957G>A | p.Arg986His | 1 | 5.5264E-06 | 1 | 1 |  |  | 35 |
|  | 53271022 | c.2959C>T | p.Pro987Ser | 1 | 5.5159E-06 | 0 | 1 |  |  | 23.9 |
|  | 53271010 | c.2971G>C | p.Gly991Arg | 2 | 1.0989E-05 | 0 | 2 |  |  | 33 |
|  | 53268410 | c.3082G>A | p.Val1028Met | 2 | 9.7532E-06 | 0 | 2 | US | 426422 | 33 |
|  | 53268392 | c.3100C>T | p.Leu1034Phe | 1 | 5.4586E-06 | 0 | 1 |  |  | 29.1 |
|  | 53268377 | c.3115T>C | p.Tyr1039His | 1 | 5.4638E-06 | 0 | 1 |  |  | 24.5 |
|  | 53267449 | c.3155G>A | p.Gly1052Asp | 1 | 4.5695E-05 | 0 | 1 |  |  | 32 |
|  | 53267438 | c.3166A>G | p.Lys1056Glu | 1 | 5.4759E-06 | 0 | 1 |  |  | 23.6 |
|  | 53267432 | c.3172C>A | p.Leu1058Ile | 1 | 5.4791E-06 | 0 | 1 |  |  | 26.8 |
|  | 53267426 | c.3178A>G | p.Ile1060Val | 5 | 2.7405E-05 | 3 | 2 |  |  | 9.135 |
|  | 53267398 | c.3206G>A | p.Arg1069Gln | 1 | 5.5455E-06 | 0 | 1 | LP | 975246 | 35 |
|  | 53267375 | c.3229C>T | p.Arg1077Cys | 2 | 1.1407E-05 | 1 | 1 | US | 871790 | 33 |
|  | 53267362 | c.3242C>T | p.Ala1081Val | 2 | 1.036E-05 | 1 | 1 |  |  | 27.1 |
|  | **GRCh38** |  |  |  |  |  |  |  |  |  |
| GnomAD V3.1 | 53241858 | c.2941G>A | p.Val981Met | 1 | 1.1777E-05 | 0 | 1 |  |  | 32 |
|  | 53241828 | c.2971G>C | p.Gly991Arg | 1 | 1.1764E-05 | 0 | 1 |  |  | 33 |
|  | 53239263 | c.3047T>C | p.Leu1016Ser | 1 | 1.1817E-05 | 0 | 1 |  |  | 13.29 |
|  | 53239228 | c.3082G>A | p.Val1028Met | 1 | 1.1798E-05 | 0 | 1 |  |  | 33 |
|  | 53238229 | c.3193A>G | p.Ser1065Gly | 1 | 1.1886E-05 | 1 | 0 | US | 937282 | 20.3 |
|  | 53238216 | c.3206G>A | p.Arg1069Gln | 2 | 2.3778E-05 | 1 | 1 |  |  | 35 |
|  | 53238204 | c.3218C>T | p.Thr1073Ile | 1 | 1.1908E-05 | 0 | 1 |  |  | 24.1 |
|  | 53238190 | c.3232G>A | p.Glu1078Lys | 1 | 1.1931E-05 | 0 | 1 |  |  | 33 |
|  | 53238181 | c.3241G>A | p.Ala1081Thr | 1 | 1.1914E-05 | 0 | 1 |  |  | 24 |
|  | 53238180 | c.3242C>T | p.Ala1081Val | 6 | 7.1515E-05 | 1 | 5 |  |  | 27.1 |

Supplementary Table 1: Pathogenic and Tolerated Variation in the PH domain of IQSEC2

V3.1

76,156 genomes => Data from non-V2 = 57,344 genomes

125,748 exomes + 15,708 genomes

V2.1.1
