# Supplementary material for: IQSEC2‐related encephalopathy in males due to missense variants in the pleckstrin homology domain
Source: Clin Genet. 2022 Apr 6;102(1):72–7. doi: 10.1111/cge.14136 (PMC9325495; doi:10.1111/cge.14136)
Supplement: Supplementary file 3 — Table S2: Conservation of functional domain structure in ArfGEF family. [file CGE-102-72-s002.docx]

|  | IQ-Like domain | AA | % Identity | Sec7 domain | AA | % Identity | PH domain | AA | % Identity |
| --- | --- | --- | --- | --- | --- | --- | --- | --- | --- |
| **IQSEC2** | **347-376** | **30** | **100%** | **746-939** | **194** | **100%** | **951-1085** | **135** | **100%** |
| IQSEC1 | 134-163 | 30 | 90% | 517-710 | 194 | 86% | 774-866 | 93 | 70% |
| IQSEC3 | 315-344 | 30 | 74% | 644-837 | 194 | 78% | 850-983 | 134 | 52% |
|  |  |  |  |  |  |  |  |  |  |
| PSD |  |  |  | 512-706 | 195 | 40% | **756-869** | **114** | **100%** |
| PSD4 |  |  |  | 544-736 | 193 | 32% | 776-892 | 117 | 53% |
| PSD2 |  |  |  | 260-462 | 203 | 35% | 512-625 | 114 | 72% |
| PSD3 |  |  |  | 534-734 | 201 | 36% | 785-898 | 114 | 68% |
|  |  |  |  |  |  |  |  |  |  |
| CYTH1 |  |  |  | 72-202 | 130 | 42% | **260-377** | **118** | **100%** |
| CYTH2 |  |  |  | 72-201 | 130 | 41% | 259-376 | 118 | 89% |
| CYTH3 |  |  |  | 77-206 | 130 | 41% | 264-381 | 118 | 89% |
| CYTH4 |  |  |  | 54-241 | 188 | 39% | 259-375 | 117 | 74% |
| GBF1 |  |  |  | 692-882 | 191 | 40% |  |  |  |
| ARFGEF1 |  |  |  | 709-840 | 132 | 44% |  |  |  |
| ARFGEF2 |  |  |  | 654-785 | 132 | 41% |  |  |  |
| FBXO8 |  |  |  | 146-276 | 131 | 36% |  |  |  |

Supp Table 2: Conservation of functional domain structure in ArfGEF family.
